# Supplementary material for: The Design, Synthesis, and Evaluation of Diaminopimelic Acid Derivatives as Potential dapF Inhibitors Preventing Lysine Biosynthesis for Antibacterial Activity
Source: Antibiotics (Basel). 2022 Dec 28;12(1):47. doi: 10.3390/antibiotics12010047 (PMC9854792; doi:10.3390/antibiotics12010047)
Supplement: Supplementary file 1 [file antibiotics-12-00047-s001.zip › antibiotics-2061477-supplementary.pdf]

Supplementary Materials File 1

The Design, Synthesis, and Evaluation of Diaminopimelic Acid Derivatives as Potential *dap*F Inhibitors Preventing Lysine Biosynthesis for Antibacterial Activity

Mohd Sayeed Shaikh [1,*], Mayura A. Kale [2], V. Muralidharan [3], T. Venkatachalam [4], Syed Sarfaraz Ali [5], Fahadul Islam [6], Sharuk L. Khan [7], Falak A. Siddiqui [7], Humaira Urmee [8], Ganesh G. Tapadiya [1], Sachin S. Dhawale [1], Long Chiau Ming [9], Ibrahim Abdel Aziz Ibrahim [10], Abdullah R. Alzahrani [10], Md. Moklesur Rahman Sarker [11,12,*] and Mohd Fahami Nur Azlina [13,*]

[1] Shreeyash Institute of Pharmaceutical Education and Research, Aurangabad 431005, Maharashtra, India; ganeshtapadiya@gmail.com (G.G.T.); sdhawale021@gmail.com (S.S.D.)
[2] Government College of Pharmacy, Aurangabad 431005, Maharashtra, India; kale_mayura@yahoo.com
[3] Vishnu Institute of Pharmaceutical Education and Research, Narsapur 502313, Hyderabad, India; vmd1213@gmail.com
[4] JKKMMRFs-Amnai JKK Sampoorani Ammal College of Pharmacy, Erirmedu, Kumarapalaiyam 638183, Tamil Nadu, India; venkatachalammpharm@yahoo.co.in
[5] Sub District Hospital, Ambad, Dist. Jalna 431204, Maharashtra, India; ss100pharma@gmail.com
[6] Department of Pharmacy, Faculty of Allied Health Sciences, Daffodil International University,
Dhaka 1207, Bangladesh; fahadulislamdiu@gmail.com
[7] Department of Pharmaceutical Chemistry, N.B.S. Institute of Pharmacy, Ausa 413520, Maharashtra, India; sharique.4u4@gmail.com (S.L.K.); falakarjumand26@gmail.com (F.A.S.)
[8] Department of Pharmaceutical Science, North South University, Dhaka 1229, Bangladesh; humaira.urmee101@gmail.com
[9] PAP Rashidah Sa'adatul Bolkiah Institute of Health Sciences, Universiti Brunei Darussalam, Gadong BE1410, Brunei; longchiauming@gmail.com
[10] Department of Pharmacology and Toxicology, Faculty of Medicine, Umm Al-Qura University, Makkah 21421, Saudi Arabia; iamustafa@uqu.edu.sa (I.A.A.I.); aralzahrani@uqu.edu.sa (A.R.A.)
[11] Department of Pharmacy, State University of Bangladesh, 77 Satmasjid Road, Dhanmondi, Dhaka 1205, Bangladesh
[12] Health Med Science Research Limited, 3/1, Block F, Lalmatia, Dhaka 1207, Bangladesh
[13] Department of Pharmacology, Faculty of Medicine, University Kebangsaan Malaysia, Jalan Yacob Latif, Kuala Lumpur 56000, Malaysia
* Correspondence: mohdsayeedsk@outlook.com (M.S.S.); dr.moklesur2014@gmail.com (M.M.R.S.);
nurazlinamf@ukm.edu.my (M.F.N.A.)

Table of Contents

1. General methods 3

2. Experimental procedure and product analysis 3

3. FT-IR and Maldi TOF-MS spectra of products 9

1. General methods

All starting materials and reagents were purchased from Dipa Chemical Laboratory Aurangabad and used without further purification. The solvents used were either of analytical grades or dried and distilled immediately prior to use. All the reactions were performed using oven-dried glassware. Melting points were recorded on a VEGO Electrothermal digital melting point apparatus and are uncorrected. Microwave-assisted organic synthesis (MAOS) was carried out in scientific catalytic microwave system CATA-RI on the 325 Watt (50% power). TLC was performed using Merck pre-coated Silica gel 60 F254 aluminum sheets (20 × 20 cm, layer thickness 0.2 mm) and Merck pre-coated Silica gel RP-C18 F254 aluminum sheets (20 × 20 cm, layer thickness 0.2 mm) and spots were visualized by UV (wavelength - 254 nm) after placing in iodine chamber. All reaction products were stored in refrigerator at around 4º C. Elemental analysis (% C, H, N) is carried out by a Perkin-Elmer 2400 CHN analyzer. IR (Infrared) spectra of all compounds have been recorded on a Prestige FT-IR spectrophotometer in KBr. Mass spectra have been scanned on a Maldi TOF-MS spectrometer.

2. Experimental procedure and product analysis

Synthesis of 2-amino-3-(1,3-thiazole methyl salfanyl)-propionic acid (1) (Thio-DAP)

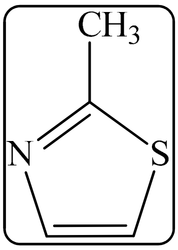

2-methylthiazole (5)

Synthesis of **2-methylthiazole (5)** was carried out by mixing of thioacetamide (3 mmol) in DMF (10 mL) and 2-chloroacetone (3 mmol) in ethanol (10 mL) was mixed in 250ml round bottom flask containing a magnetic stirrer bar and refluxed for 3.5 hours at 80^0 C with stirring. The completion of reaction was checked by Thin Layer Chromatography (TLC) using silica gel G as stationary phase employing toluene, ethyl acetate and formaldehyde (4:4:2) as solvent system. The reaction mixture was cooled to room temperature and poured into ice-cold water (50 mL) to get the solid product 2-methylthiazole (5). The resulting precipitate was collected by filtration and recrystallized from ethanol-DMF mixture to purify the compound. The results showed that maximum yield of 2-methylthiazole (5) (80%).

White liquid; Molecular Formula-C_4H_5NS; Molecular Weight-99.15; % Yield-73.69; Bp: 129-130 ºC; Rf 0.61; FTIR (KBr disk)- 881.47, 750.31, (Aromatic C-H str. out of plane bend), 937.40 (Alkene C-H str. out of plane bend)), 1008.77, 1118.71, 1217.08 (>C-N- str.), 1033.85 (>C-O- str.), 1433.11, 1333.81 (-CH_3 bending), 1550.77 (>C=C< Aromatic conjugation), 1666.50 (>C=N- str), 2870.35 (C-H Alkanes str.), 3138.18 (C-H aromatic) cm-1; Anal. Calcd. For Molecular Formula-C_4H_5NS: C-(48.45 %), H-(5.08 %), N-(14.13 %); S-(32.34 %).

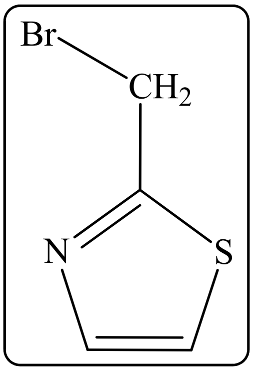

2-(bromomethyl)thiazole (7)

A solution of 2-Methyl thiazole (10 mmol), N -bromosuccinimide (10 mmol) and dibenzoyl peroxide (1 mmol) in 60 mL of dry carbon tetrachloride was refluxed for 8 h, cooled to room temperature, and then filtered. The filtrate was concentrated under reduced pressure to afford the product. Trituration of the product with diethyl ether furnished 2-Bromo-methyl thiazole as a white solid in 92% yield.

Light yellow solid; Molecular Formula- C_4H_4NSBr; Molecular Weight- 179.054; % Yield- 66.91; Bp: 150 -153 ºC; Rf 0.42; FTIR (KBr disk)- 667.37, 513.07 (C-Br), 883.40 (Aromatic C-H str. out of plane bend), 1031.92 (>C-N- str.), 1215.15 (>C-O- str.), 1415.75 (-CH_3 bending), 1556.55 (>C=C< Aromatic conjugation), 1666.50 (>C=N- str.), 1940.39, 2009.83, 2115.91 (-S-C-N-), 2983.88 (C-H Alkanes str.), 3317.56 (C-H aromatic) cm-1; Anal. Calcd. For Molecular Formula- C_4H_4NSBr: C-(26.98 %), H-(2.26 %), N-(7.87 %); S-(18.01 %), Br-(44.88).

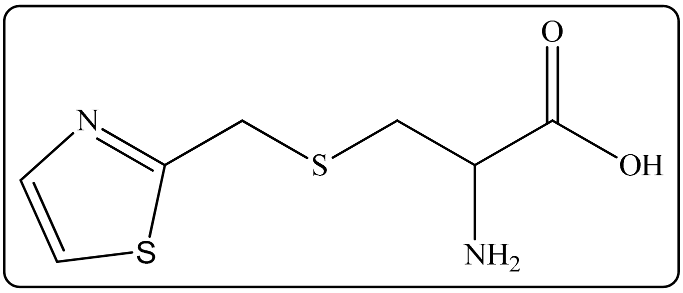

2-amino-3-(1,3-thiazole methyl salfanyl)-propionic acid (1) (Thio-DAP)

K_2CO_3 (10 mmol) was added portion-wise to a solution of L-cystiene (2 mmol) in acetone (70 mL). The mixture was stirred for 0.5 h at the refluxing temperature. Compound 2-Bromo-methyl thiazole (3mmol) was then added to the solution, which was refluxed for 45 minutes in scientific catalytic microwave system CATA-RI on the 325 Watt (50% power. After the solvent was evaporated under vacuum, the residue was chromatographed on silica gel with petroleum ether/ethyl acetate as eluent. An analytical sample was recrystallized from petroleum ether/ethyl acetate.

White crystalline solid; Molecular Formula- $C_7H_{10}N_2O_2S_2$; Molecular Weight- 219.31 gm/mol; % Yield- 69.31; Mp: 192-196 ºC; Rf 0.71; FTIR (KBr disk)- 867.97, 821.68, 769.60 (Aromatic C-H out of plane bend), 943.19 (Alkene C-H out of plane bend), 1064.71, 1001.06 (>C-N- Stretch), 1300.00, 1195.87 (>C-O- Stretch), 1350.17 ($-CH_3$ bending), 1516.05 (>C=C< Aromatic conjugation), 1647.21 (>C=N- Stretch), 1762.54 (>C=O Carboxylic acid), 2108.20, 2009.83 (S-C-N cyclic, thiazole ring), 2866.22 (C-H Alkanes Stretch), 3317.56 (C-H aromatic Stretch), 3264.35 (O-H aromatic Stretch), 3007.75 (N-H Stretch Primary Amine) cm-1; Anal. Calcd. For Molecular Formula- $C_7H_{10}N_2O_2S_2$: C-(38.51 %), H-(4.62 %), N-(12.83 %); O-(14.66), S-(29.38 %). Maldi TOF-MS: m/z values, 100.125 (100.0%), 130.583 (36.54%), 136.054 (19.49%), 220.019 (33.58.35%) (M+1).

Synthesis of 2-amino-3-(1,3-oxazole methyl salfanyl)-propionic acid (2) (Oxa-DAP)

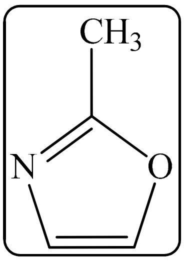

2-methyloxazole (11)

Synthesis of **2-methyloxazole (11)** was carried out by mixing of acetamide (5 mmol) in DMF (10 mL) and 2-chloroacetone (5 mmol) in ethanol (10 mL) was mixed in 250ml round bottom flask containing a magnetic stirrer bar and refluxed for 4.5 hours at 80^0 C with stirring. The completion of reaction was checked by Thin Layer Chromatography (TLC) using silica gel G as stationary phase employing toluene, ethyl acetate and formaldehyde (4:4:2) as solvent system. The reaction mixture was cooled to room temperature and poured into ice-cold water (50 mL) to get the solid product 2-methylthiazole (5). The resulting precipitate was collected by filtration and

recrystallized from ethanol-DMF mixture to purify the compound. The results showed that maximum yield of 2-methyloxazole (11) (71 %).

White crystalline solid; Molecular Formula-C_4H_5NO; Molecular Weight-83.09; % Yield-59.19; Bp: 87-90 ºC; Rf 0.45; FTIR (KBr disk)- 891.11, 825.93, 763.81 (Aromatic C-H out of plane bend), 941.26 (Alkene C-H out of plane bend), 1220.94, 1118.71 (>C-N- str.), 1033.85 (>C-O), 1421.54, 1334.74 ($-CH_3$ bending), 1556.55 (>C=C< (Aromatic conjugation)), 1703.14 (>C=N- str.), 2935.25 (C-H Alkanes str.), 3196.05 (C-H aromatic) cm-1; Anal. Calcd. For Molecular Formula-C_4H_5NO: C-(57.82 %), H-(6.07 %), N-(16.86 %); O-(19.26 %).

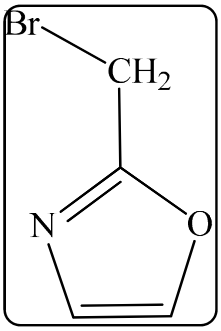

2-(bromomethyl)oxazole (12)

A solution of 2-Methyl thiazole (10 mmol), N -bromosuccinimide (10 mmol) and dibenzoyl peroxide (1 mmol) in 60 mL of dry carbon tetrachloride was refluxed for 8 hours, cooled to room temperature, and then filtered. The filtrate was concentrated under reduced pressure to afford the product. Trituration of the product with diethyl ether furnished **2-(bromomethyl)oxazole** as a white solid in 92% yield.

White solid; Molecular Formula- C4H4BrNO; Molecular Weight- 161.98; % Yield- 71.52; Bp: 173 – 175 ºC; Rf 0.37; FTIR (KBr disk)- 891.11, 825.93, 763.81 (Aromatic C-H out of plane bend), 1107.14, 1024.20 (>C-N), 1207.44 (>C-O), 1402.25 ($-CH_3$ bending), 1556.55 (>C=C< (Aromatic C-H conjugation), 1712.79 (>C=N- str.), 2945.35 (C-H Alkanes str.),

3196.05 (C-H aromatic) cm-1; Anal. Calcd. For Molecular Formula- C4H4BrNO: C-(29.66 %), H-(2.49 %), N-(8.65 %); O-(9.88 %), Br-(49.33).

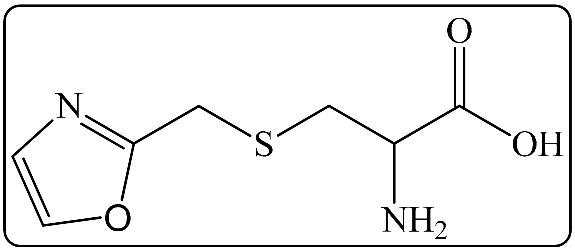

3-((oxazol-2-yl)methylthio)-2-aminopropanoic acid (02) (Oxa-DAP)

K_2CO_3 (10 mmol) was added portion-wise to a solution of L-cystiene (2 mmol) in acetone (70 mL). The mixture was stirred for 0.5 h at the refluxing temperature. Compound **2-(bromomethyl)oxazole** (3mmol) was then added to the solution, which was refluxed for 45 minutes in scientific catalytic microwave system CATA-RI on the 325 Watt (50% power. After the solvent was evaporated under vacuum, the residue was chromatographed on silica gel with petroleum ether/ethyl acetate as eluent. An analytical sample was recrystallized from petroleum ether/ethyl acetate.

White crystalline solid; Molecular Weight- 202.23 gm/mol; Molecular formula- $C_7H_{10}N_2O_3S$; % Yield-73.32; Mp: 215-217 ºC; Rf 0.61; FTIR (KBr disk) 869.90, 821.68, 771.53 (Aromatic C-H out of plane bend), 3061.03, 2544.11 (N-H Stretch Primary Amine), 943.19 (Alkene C-H out of plane bend), 1001.06 (N-C-O cyclic, oxazole ring), 1064.71 (C-N amine), 1195.87 (>C-O Stretch), 1300.00 (N-C-O oxazole ring Stretch), 1425.40, 1352.10 (-CH_3 bending), 1517.98 (>C=C< Aromatic conjugation), 1649.14 (>C=N- Stretch), 1743.32 (C=O Carboxylic acid), 2083.12 (O-C-N cyclic oxazole ring), 2866.22 (C-H Alkanes Stretch), 3343.50 (O-H aromatic Stretch) cm^{-1}; Anal. Calcd. For $C_7H_{10}N_2O_3S$: C (41.57%), H (4.98%), N (13.85%), O (23.73%), S (15.86%); Maldi TOF-MS: m/z values, 83.735 (100.0%), 115.535 (20.15%), 159.325 (14.57%), 203.121 (21.03.35%).

3. FT-IR and Maldi TOF-MS spectra of products

2-methylthiazole (5)

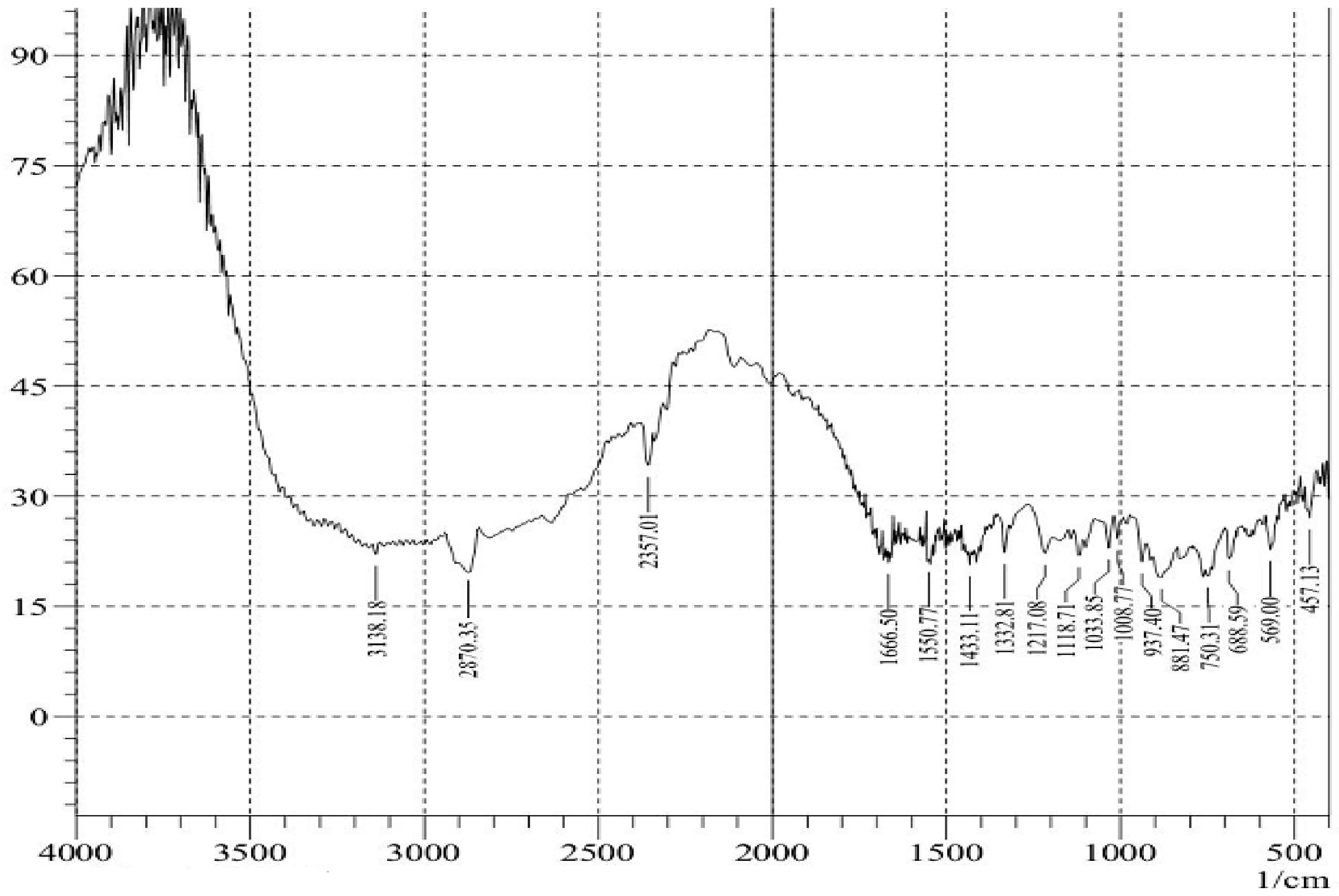

2-(bromomethyl)thiazole (7)

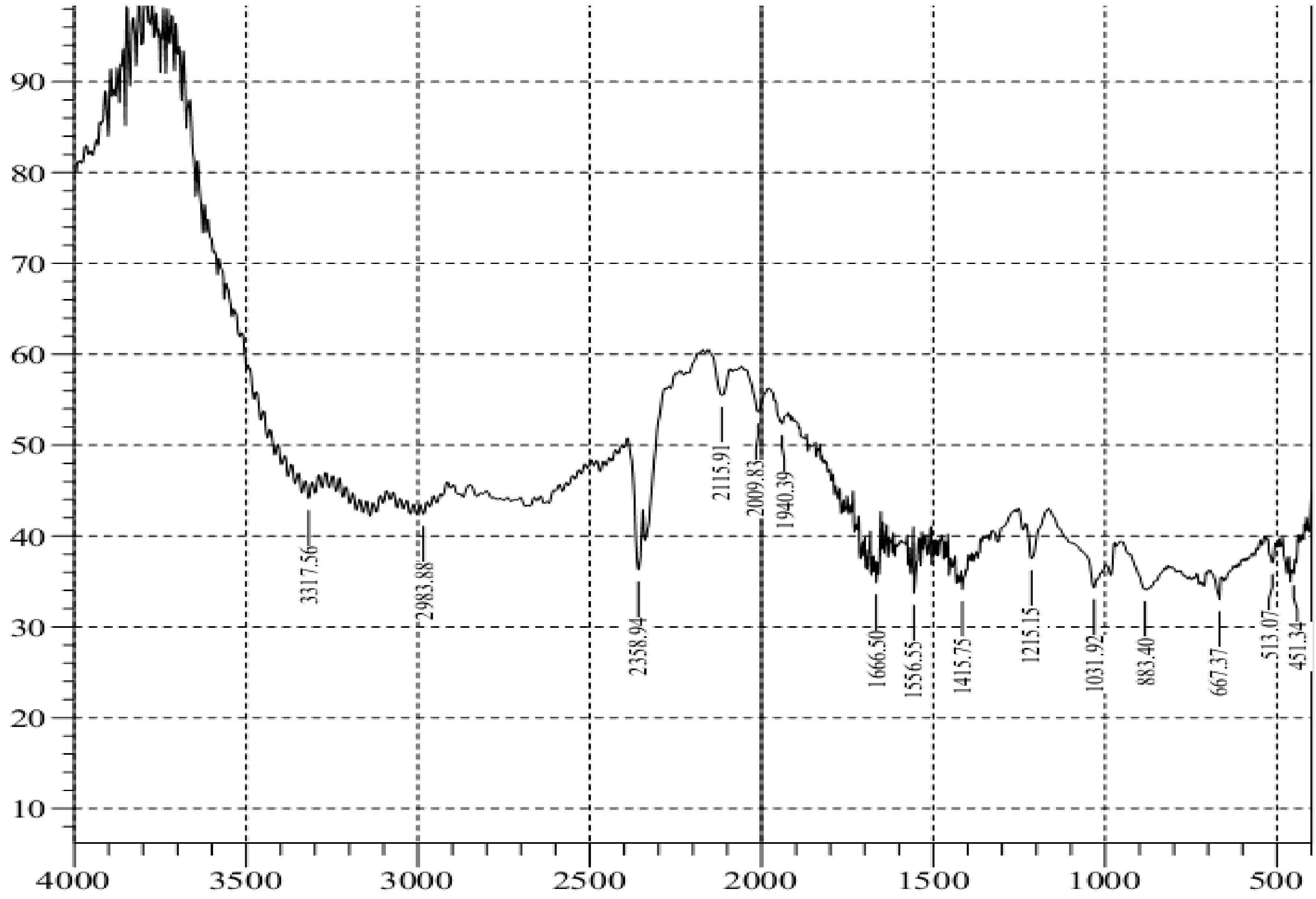

2-amino-3-(1,3-thiazole methyl salfanyl)-propionic acid (1) (Thio-DAP)

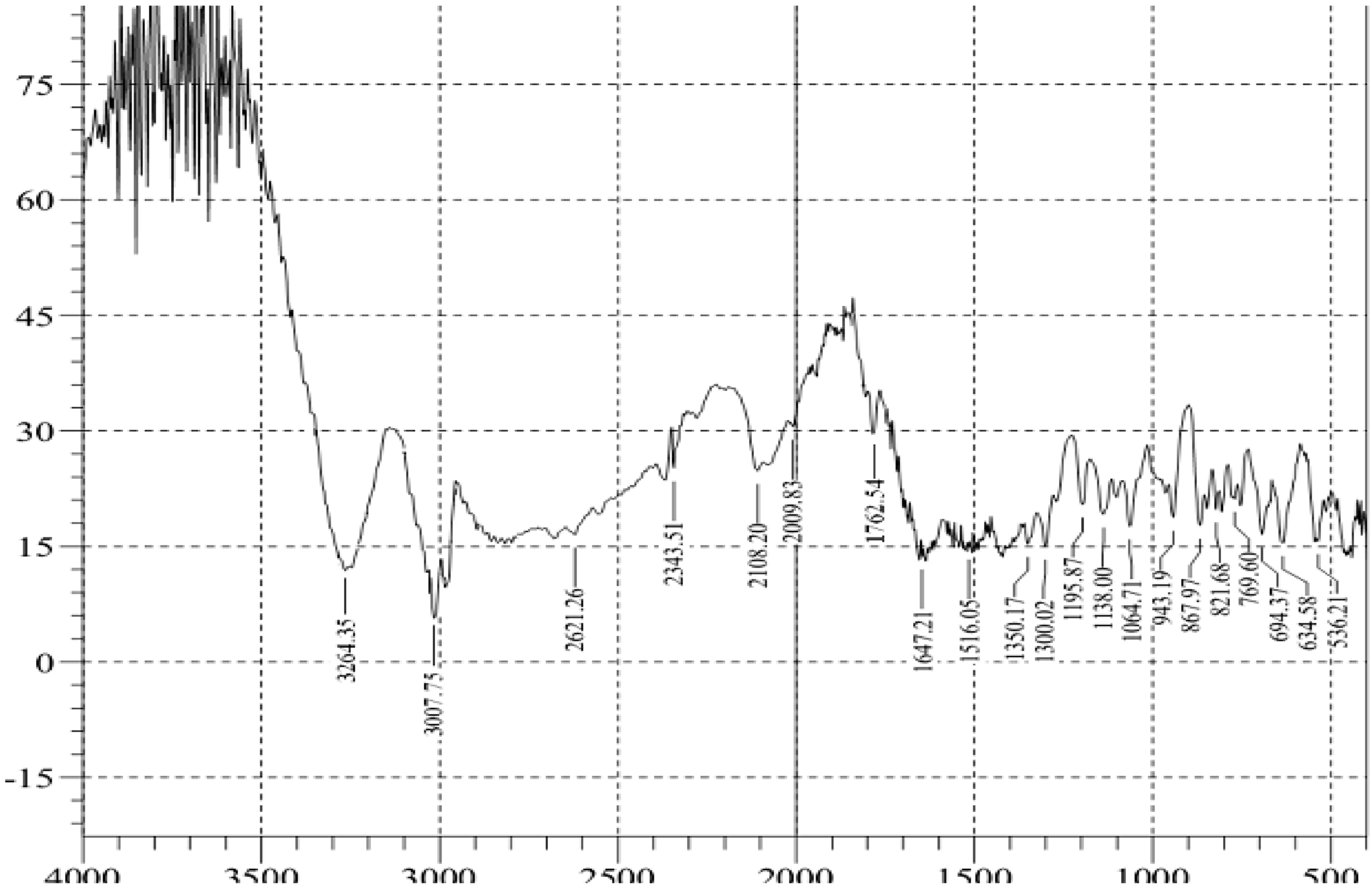

2-methyloxazole (11)

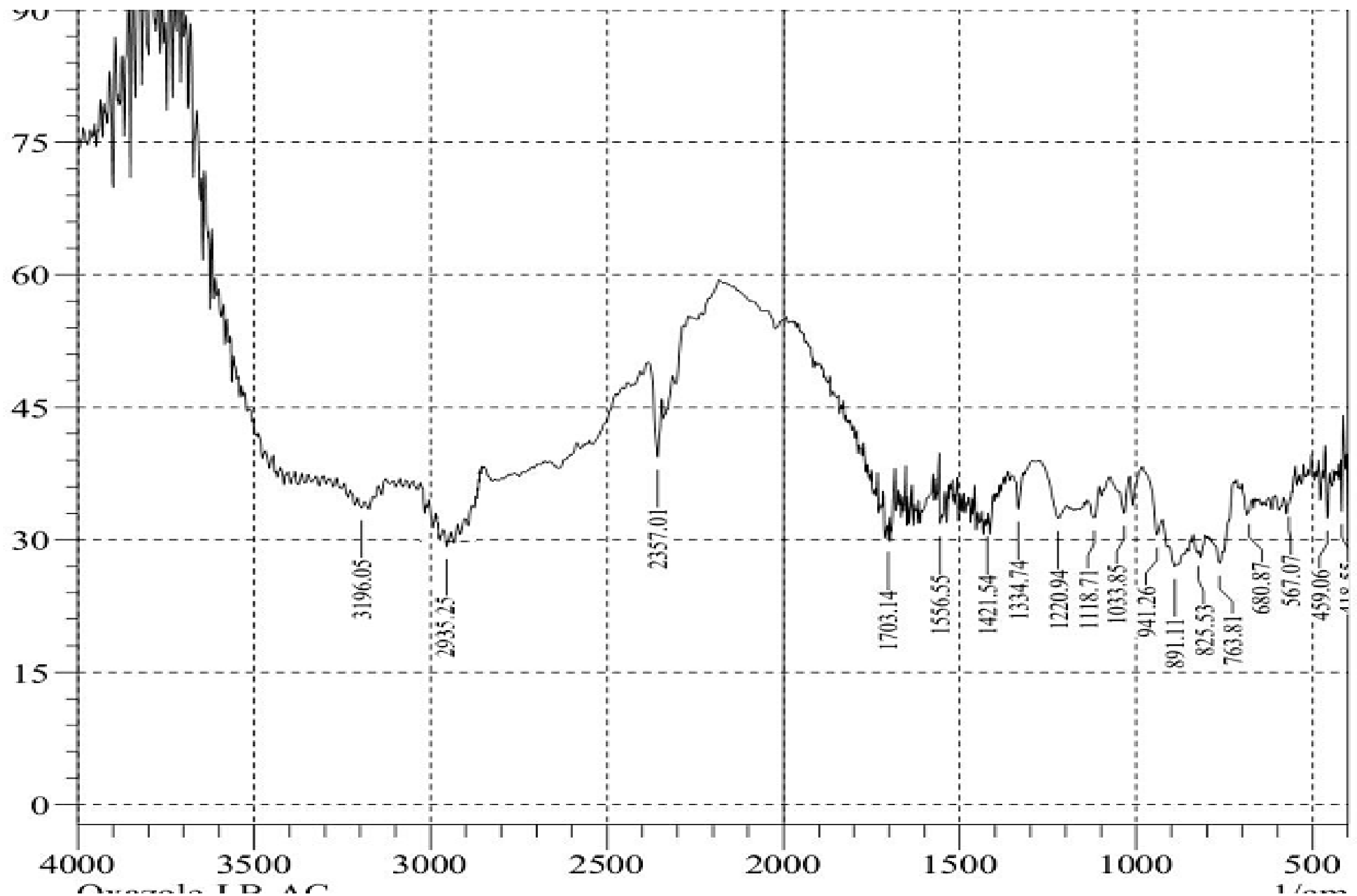

2-(bromomethyl)oxazole (12)

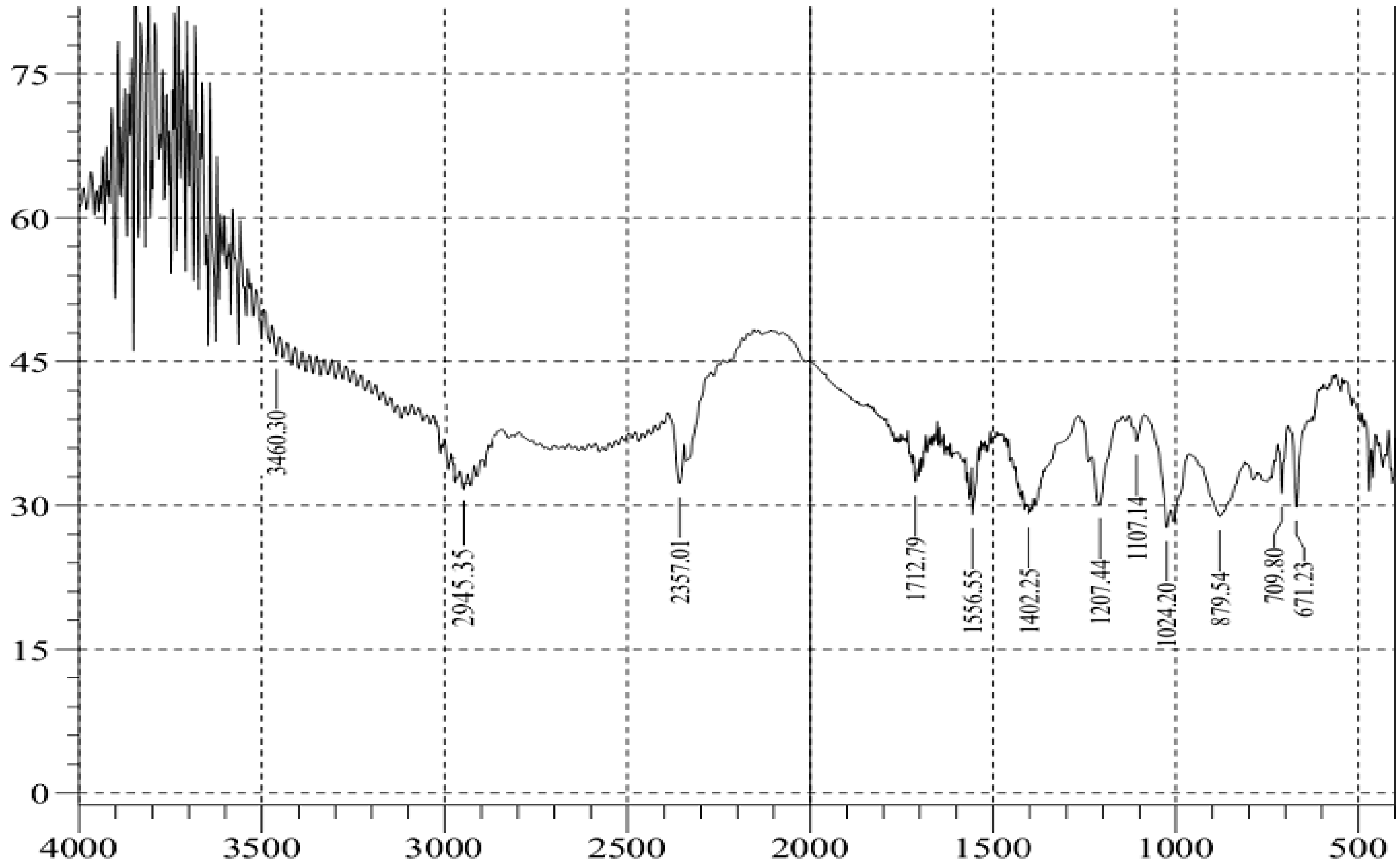

3-((oxazol-2-yl)methylthio)-2-aminopropanoic acid (02) (Oxa-DAP)

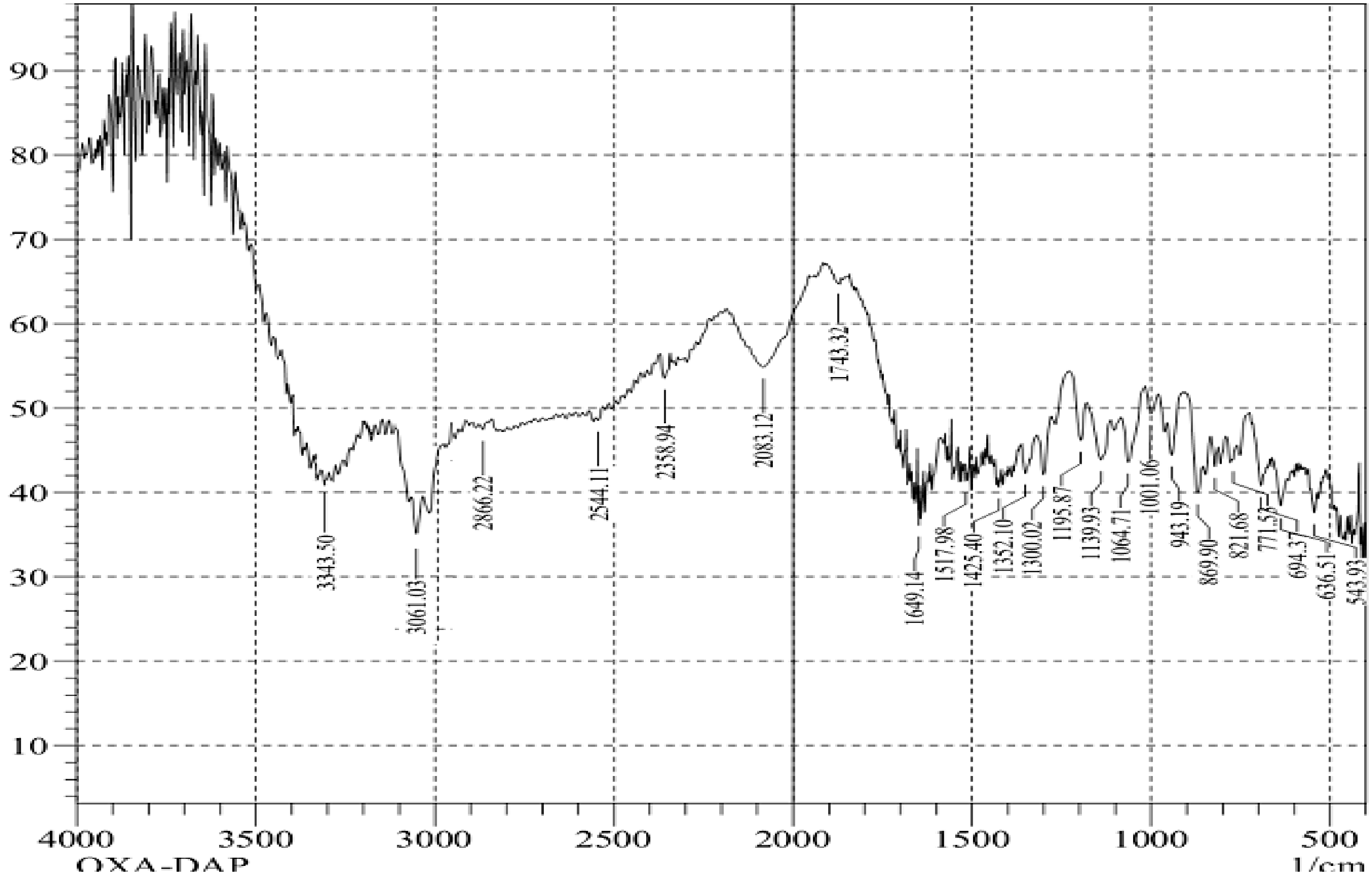

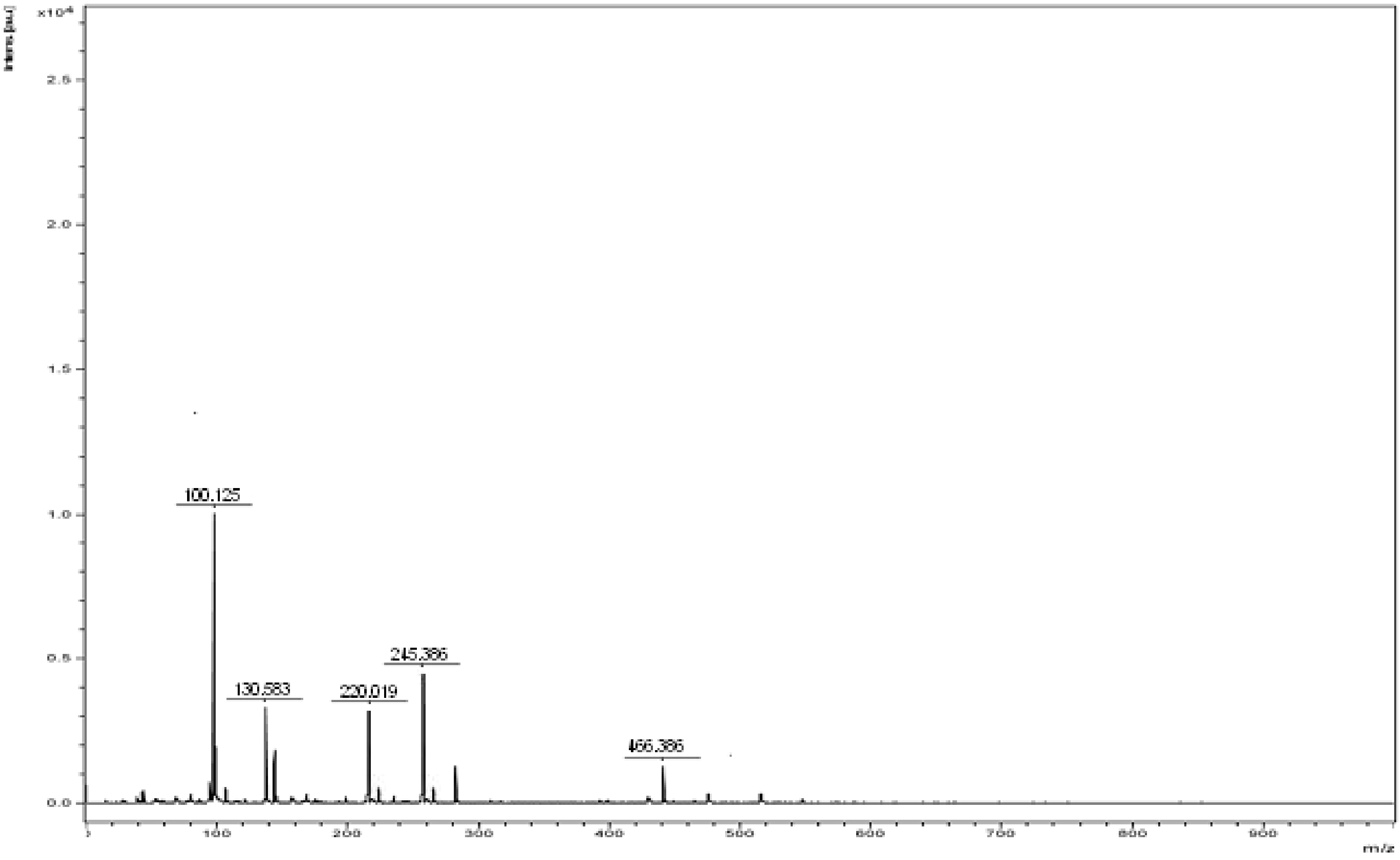
x10^4
Intens. [a.u.]
2.5
2.0
1.5
1.0
0.5
0.0
100.125
130.583
220.019
245.386
466.386
0
100
200
300
400
500
600
700
800
900
m/z

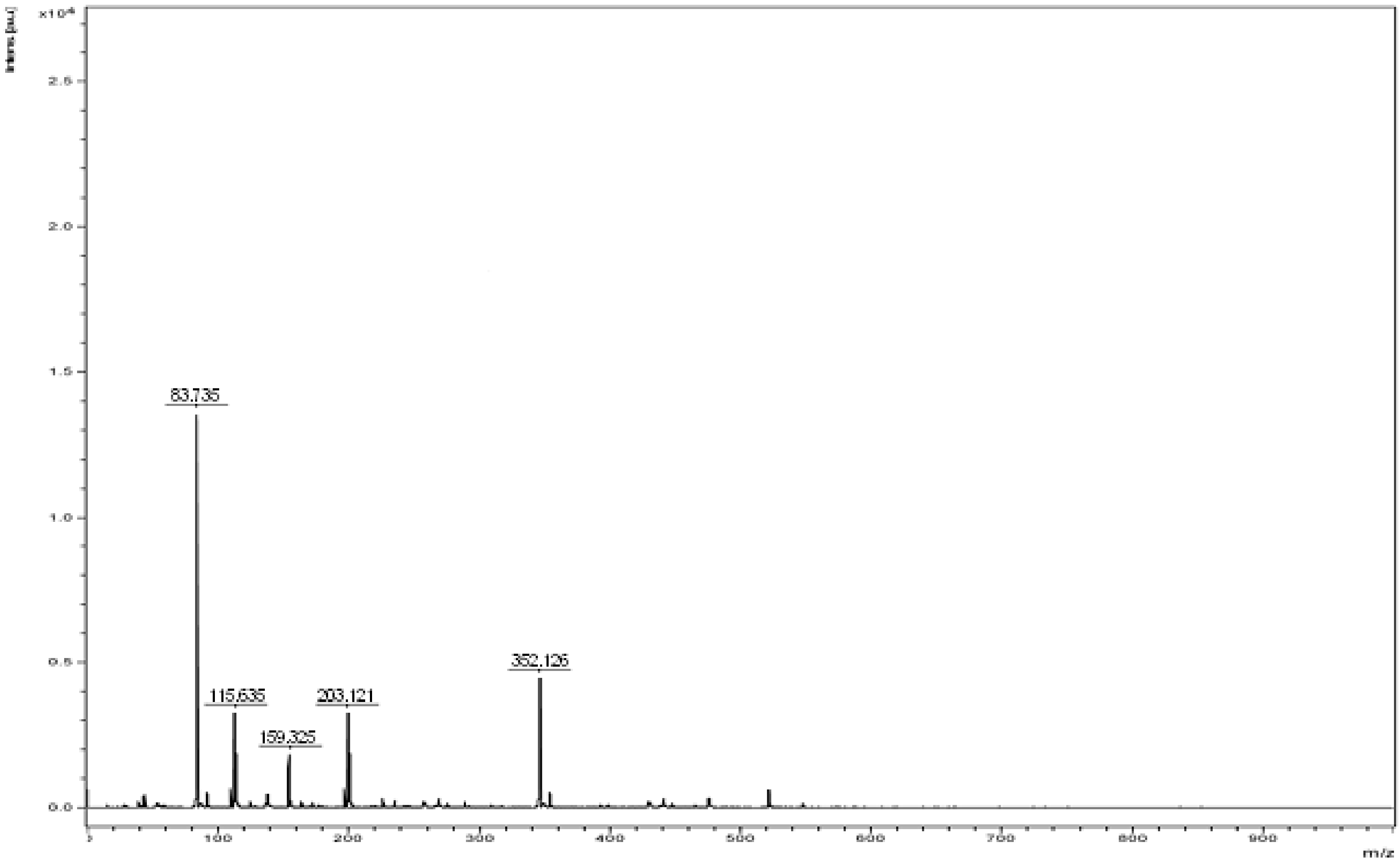

Intens. [a.u.]
x10⁴
2.5
2.0
1.5
1.0
0.5
0.0
83.735
115.635
159.325
203.121
352.126
100
200
300
400
500
600
700
800
900
m/z
